# Effects of marine actinomycete on the removal of a toxicity alga *Phaeocystis globose* in eutrophication waters

**DOI:** 10.3389/fmicb.2015.00474

**Published:** 2015-05-19

**Authors:** Huajun Zhang, Su Zhang, Yun Peng, Yi Li, Zhangran Chen, Hong Xu, Zhiming Yu, Wei Zheng, Tianling Zheng

**Affiliations:** ^1^State Key Laboratory of Marine Environmental Science and Key Laboratory of the Ministry of Education for Coastal and Wetland Ecosystems, School of Life Sciences, Xiamen UniversityXiamen, China; ^2^Key Laboratory of Marine Ecology and Environmental Science, Institute of Oceanology, Chinese Academy of SciencesQingdao, China

**Keywords:** *Phaeocystis globose*, algicidal actinomycetes, membrane permeability, photosynthetic activity, gene expression

## Abstract

*Phaeocystis globosa* blooms in eutrophication waters can cause severely damage in marine ecosystem and consequently influence human activities. This study investigated the effect and role of an algicidal actinomycete (*Streptomyces* sp. JS01) on the elimination process of *P. globosa*. JS01 supernatant could alter algal cell membrane permeability in 4 h when analyzed with flow cytometry. Reactive oxygen species (ROS) levels were 7.2 times higher than that at 0 h following exposure to JS01 supernatant for 8 h, which indicated that algal cells suffered from oxidative damage. The Fv/Fm value which could reflect photosystem II (PS II) electron flow status also decreased. Real-time PCR showed that the expression of the photosynthesis related genes *psbA* and *rbcS* were suppressed by JS01 supernatant, which might induce damage to PS II. Our results demonstrated that JS01 supernatant can change algal membrane permeability in a short time and then affect photosynthesis process, which might block the PS II electron transport chain to produce excessive ROS. This experiment demonstrated that *Streptomyces* sp. JS01 could eliminate harmful algae in marine waters efficiently and may be function as a harmful algal bloom controller material.

## Introduction

The public health, tourism, fisheries, and ecosystem impacts from HABs have all increased over the past few decades because of the continuous increase in nutrients, especially nitrogen and phosphorus. HABs which generally indicate eutrophication in marine coastal waters occur worldwide not only in coastal marine ecosystems but also in open ocean (Anderson, 1997; Anderson et al., 2012). *Phaeocystis* (Prymnesiophyceae) is an important bloom-forming phytoplankton which is a globally distributed genus. *Phaeocystis* blooms can draw down atmospheric CO_2_ and produce dimethylsulphide (DiTullio et al., 2000; Brussaard et al., 2005; Hoogstraten et al., 2012), which have an impact on the chemical quality of the atmosphere and global climate regulation. HABs dominated by *Phaeocystis globosa* are recurrent events in marine ecosystems (Lamy et al., 2009). *P. globosa* regularly dominates the phytoplankton community in the coastal waters, while its blooms may result in “stinking water” and the production of foam of mucilaginous material that deposit on beaches (Baudoux and Brussaard, 2005). This species has a heteromorphic life cycle, forming flagellated solitary cells as well as colonies.

Frequent HAB outbreaks in marine waters have led to heightened scientific and regulatory attention, and the development of many new technologies and approaches for research and management (Anderson et al., 2012). The use of biological control agents including bacteria, actinomycetes, and viruses are among the most widely studied methods for groundwater and marine water treatment (Wang et al., 2010a; Santini et al., 2013; Zhang et al., 2013a; Chen et al., 2014). Studies on the relationship between algae and bacteria have resulted in the isolation of actinomycetes capable of inhibiting or killing HAB species. The reported algicidal actinomycetes are mostly *Streptomyces* species (Choi et al., 2005). Actinomycetes can produce various bioactive substances and are considered as potential and effective biological agents to eliminate HAB species in eutrophication waters.

Studies imply that extracellular substances secreted by algicidal actinomycetes play an important role in algicidal activity and this is called allelopathy. It is now becoming important to understand how algae respond to these allelochemicals. The physiological and biochemical responses of microalgae to allelochemical stresses have been extensively studied. Aquatic organisms including algae could eliminate ROS according to antioxidative enzymes such as SOD, CAT, and peroxidases to avoid oxidative damage (Leitão et al., 2003; Yang et al., 2011; Li et al., 2014a). FCM is a rapid method for the quantitative measurement of individual cells in a moving fluid. This technique can analyze multiparameters on a wide range of cellular properties by determining algal light-scatter signals and autofluorescence. FCM can also provide more information regarding the physiological condition of cells using biochemically specific fluorescent dyes (Faber et al., 1997; Liu et al., 2008). FCM offers the advantage of being able to measure the intracellular fluorescence of cells stained with propidium iodide (PI) which can be used to analyze algal cell membrane permeability.

The present work was undertaken to determine the effect of an algicidal actinomycetes *Streptomyces* sp. JS01 on the harmful alga *P. globosa.* In order to illustrate the algal lysis process of JS01, further studies were carried out: (1) to investigate algal cell membrane permeability by using FCM; (2) to explore the effect of JS01 on the PS II of algal cells; (3) to study the oxidative stress in algal cells induced by JS01; and (4) to observed algicidal process under transmission electron microscopy (TEM).

## Materials and Methods

### Algicidal Components Preparation

*Streptomyces* sp. JS01 which had been deposited in MCCC (Marin Culture Collection of China) with the accession number of MCCC 1F01225 was isolated from the coastal surface water of the Xiamen Sea. The 16S rRNA gene sequence had been deposited in NCBI database with GenBank accession number KM657967. Cells of JS01 were inoculated into Zobell 2216E broth (peptone 5 g/L, yeast extract 1 g/L, ferric phosphorous acid 0.1 g/L, dissolved in natural seawater, pH7.6–7.8) followed by incubation for 6 days at 28∘C. Then the cells were removed using centrifugation at 10,000 ×*g* for 10 min and the supernatant was filtered through a 0.22 μm Millipore membrane. The supernatant was then stored at –80∘C.

### Algal Growth and JS01 Supernatant Treatment

Cultures of experimental alga, *P. globosa*, were supplied by the State Key Laboratory of Marine Environmental Science (Xiamen University). The cultures were incubated in sterile f/2 medium (without silicate) prepared with natural seawater at 20 ± 1∘C under a 12 h: 12 h light-dark cycle with a light intensity of 50 μmol photons m^-2^s^-1^(Su et al., 2007). To analyze the pigments content of chlorophyll *a* (Chl *a*), 5 mL algal cultures were collected, and washed with PBS (50 mM, pH7.8); Then pigments were extracted using 90% ethanol in the dark overnight at 4∘C (Inskeep and Bloom, 1985). After centrifugation at 12,000 × *g* for 5 min, we measured the supernatant absorbance values at wavelengths of 665, 645 nm. Algal growth rates was detected every day using Chl *a* content and calculated by the formula below.

(1) $$Chlorophyll\,\;a\,\;(mg/L)=12.7*A_{665}\;-2.69*A_{645}$$

Where A_665_ and A_645_ represent absorbance values at wavelengths of 665, 645 nm, respectively.

JS01 supernatant (prepared as described above) were added into 100 mL axenic exponentially growing algal cultures at a ratio of 3.5% (v/v) in triplicate and the same volume of 2216E broth was also added into algal cultures serving as control.

### Algicidal Effects of JS01 against *P. globosa*

JS01 supernatant were added into 100 mL axenic exponentially growing algal cultures at a ratio of 1.5, 3.5, and 5.5% in triplicate in order to measure the algicidal rate based on the removal rate of Chl *a* using the formula below (Inskeep and Bloom, 1985). Autoclaved Zobell 2216E broth served as the control.

(2) $$Chlorophyll\,\;a\,\;removal\,\;rate\,\;\left(\%\right)\,\;=\;\left(1-C_{T}/C_{C}\right)*100\%$$

*C*_T_ represents the content of Chl *a* in the treatment group and and *C*_C_ the amount in the control group.

### Fluorescent Staining and Flow Cytometer (FCM) Analysis

Algal cells were collected using centrifugation at 5,000 × *g* for 5 min after treatment with JS01 supernatant, washed with PBS (50 mM, pH 7.4) three times, and then resuspended in it. For assessment of the cell viability and permeabilization of *P. globosa*, fluorescent dye PI (provided by Invitrogen, USA) which is specific for the staining of cellular acids was used (Spilimbergo et al., 2010; Zhang et al., 2013b). Hundred microliter of PI (100 μg/mL, dissolved in PBS, pH 7.4) were dosed into 900 μL of algal cell suspension containing about 10^6^–10^7^ cells/mL and final concentration of PI was 10 μg/mL. To complete the staining, samples were incubated for 15 min in the dark at room temperature. FCM analysis was performed using a BD LSRFortessa cell analyzer (BD, USA), equipped with an arc lamp as light source. Samples were illuminated with an excitation beam at wavelength of 488 nm. For each cell, PI fluorescence was collected with a 560–590 nm filter (FL2). Four thresholds for data acquisition were set on PI fluorescence in order to eliminate background and signals from debris. For each sample about 10,000 cells were analyzed.

### Determination of ROS Levels, Malondialdehyde Content, and Antioxidative Enzyme Assays

Intracellular ROS was detected using a fluorescent probe, 2′,7′- dichlorofluorescin diacetate (DCFH-DA), based on the reported method (Yin et al., 2005), but with some modifications. Algal cells were resuspended in 0.5 mL DCFH-DA (the final concentration in the mixture was 10 μM) and incubated at 37∘C in the dark for 1 h. After that, algal cells were washed three times with sterile f/2 medium immediately and finally resuspended with 1 mL sterile f/2 medium. The fluorescence intensity of algal cells was detected by a spectrofluorometer with excitation wavelength at 485 nm and emission wavelength at 525 nm.

After collection and washing with PBS, algal cells disruption was conducted using an Ultrasonic Cell Disruption System (NingBo Scientiz Biotechnological Co., Ltd, China; 120W, 5s : 5s, 80 times) below 4∘C. Debris was removed using centrifugation at 10,000 × *g* for 10 min at 4∘C. The supernatant was used to analyze the content of MDA (Malondialdehyde, byproduct of lipid peroxidation), and enzyme activities including SOD and CAT. All the analysis methods were carried out according to the kit’s Operation Manual from Nanjing Jiancheng Bioengineering Institute, China (Li et al., 2014b).

### Pigments and Chlorophyll Fluorescence Measurement

We collected about 5 × 10^6^ algal cells to detect the content of chl *a* and carotenoid after JS01 extract treatment. These methods have been described above. Carotenoid contents were calculated using the formulae:

(3) $$Carotinoid\,\;\left(mg/L\right)\,\;=\;(1000\,\;*\;A_{470}\,\;-\;2.05\,\;*\;C_{Chlorophyl\,\;a})/245$$

where, A_470_ represents absorbance values at wavelength of 470 nm; and C _Chlorophyll_ *_a_* represents the content of Chl *a*.

Pulse amplitude modulation (PAM) fluorescence measurements were performed using a PAM-CONTROL Fluorometer (Walz, Effeltrich, Germany). Algal cells were firstly cultured in the dark for 15 min and then measured under an actinic light of 3000 μmol photons m^-2^s^-1^ (Drábková et al., 2007). Maximum photochemical quantum yield of photosystem (Fv/Fm) is a biomass independent factor which can reflect the process of photosynthesis.

### RNA Extraction and Quantitative Real-Time PCR Analysis

Fifty milliliter of 6, 12, and 24 h treated algal cells were collected and frozen at –80∘C until RNA extraction. Total RNA was extracted using the RNAiso kit (TaKaRa Company, Dalian, China) following the manufacturer’s instructions. We used relative Real-time PCR to detect gene expression and the gene specific primers were forward primer 5′-AGTTGCTGGTTCTCTACTTTACG-3′ and reverse primer 5′-TTCCCAC TCACGACCGATG-3′ for the *psbA* gene; forward primer 5′- AAGTCTTACTGGGA AATGTGGG-3′ and reverse primer 5′-AGCAGGACGCTGAACGATG-3′ for the *rbcS* gene; and forward primer 5′-TCCGATAACGAACGAGAC-3′ and reverse primer 5′-TGACGCAAACTTCCACTT-3′ for the 18S rRNA gene. One step cycling was performed using amplification with an initial preheating step of 3min at 95∘C, 40 cycles at 95∘C for 10 s and 55∘C for 30 s. In order to normalize gene expression changes, 18S rRNA was used as a reference gene. The relative gene expression was quantified using the 2^-ΔΔCt^ method (Livak and Schmittgen, 2001).

### Sample Preparation and TEM Analysis

Algal cells were treated with JS01 supernatant for 12, 24, 36, 48, and 72h and after that they were fixed for TEM (Zhang et al., 2013a). Samples were viewed using a JEM2100HC (Japan) transmission electron microscope.

### Statistics

All data were analyzed using one-way analysis of variance (ANOVA) followed by the least significant difference test, with ^∗∗^*p* < 0.01 and ^∗^*p* < 0.05 (SPSS 18.0 for windows).

## Results

### Algal Growth Rates and Algicidal Activity of Actinomycete JS01 on *P. globosa*

**FIGURE 1:**
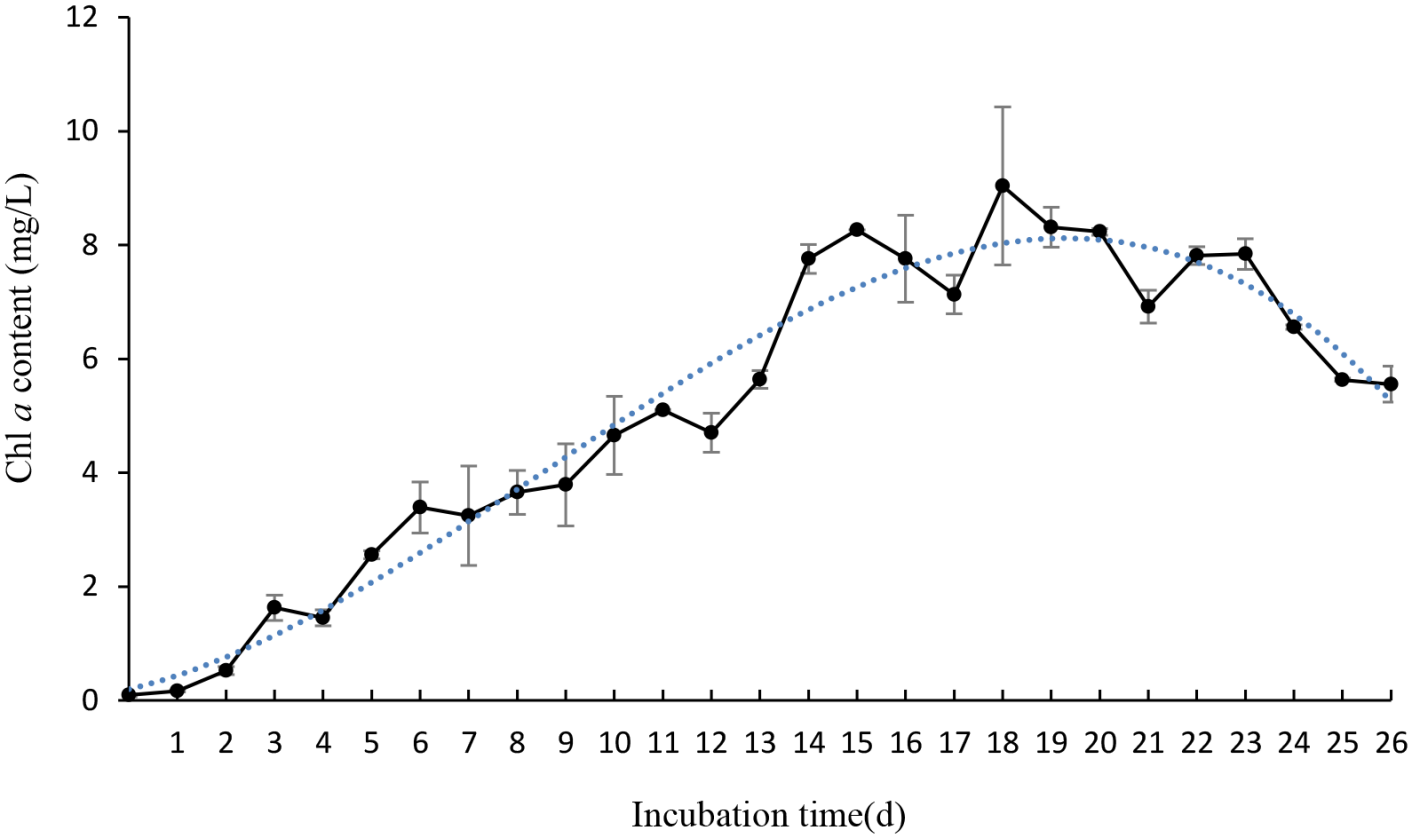
**The growth rates of *Phaeocystis globosa* when cultured in f/2 medium.** All error bars indicate SE of the three replicates.

**FIGURE 2:**
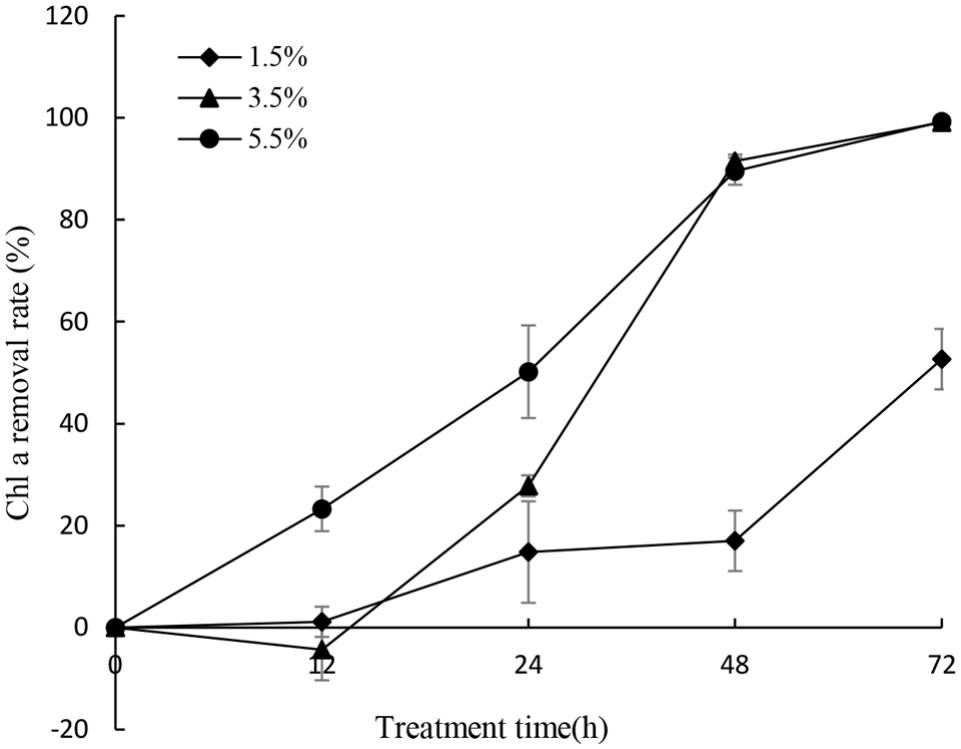
**Effect of different volume of JS01 supernatant on the Chl *a* removal rate of *P. globosa.*** All error bars indicate SE of the three replicates.

To gain efficient algicidal material, it is important to study algal growth rates in nutritionally adequate waters. **Figure 1** showed us algal growth rates of *P. globosa*. When the algae was transferred into new environment, the growth rates rose rapidly, and then dropped after 18 days. The maximum chl *a* content was about 9.03 mg/L. To determine the effective alga-lytic concentration of JS01 against *P. globosa*, different ratios of JS01 supernatant (1.5, 3.5, and 5.5%) were inoculated into algal cultures. The proportion of supernatant of 1.5% showed lower alga-lytic activity compared with the other two groups and the proportion of 3.5 and 5.5% showed a Chl *a* removal rate of 89.5% after 48 h exposure (**Figure 2**). The Chl *a* removal rate was more than 99% after 72 h treatment with the JS01 supernatant in the 3.5 and 5.5% concentrations.

### Algal Cell Membrane Permeabilization Analysis

**FIGURE 3:**
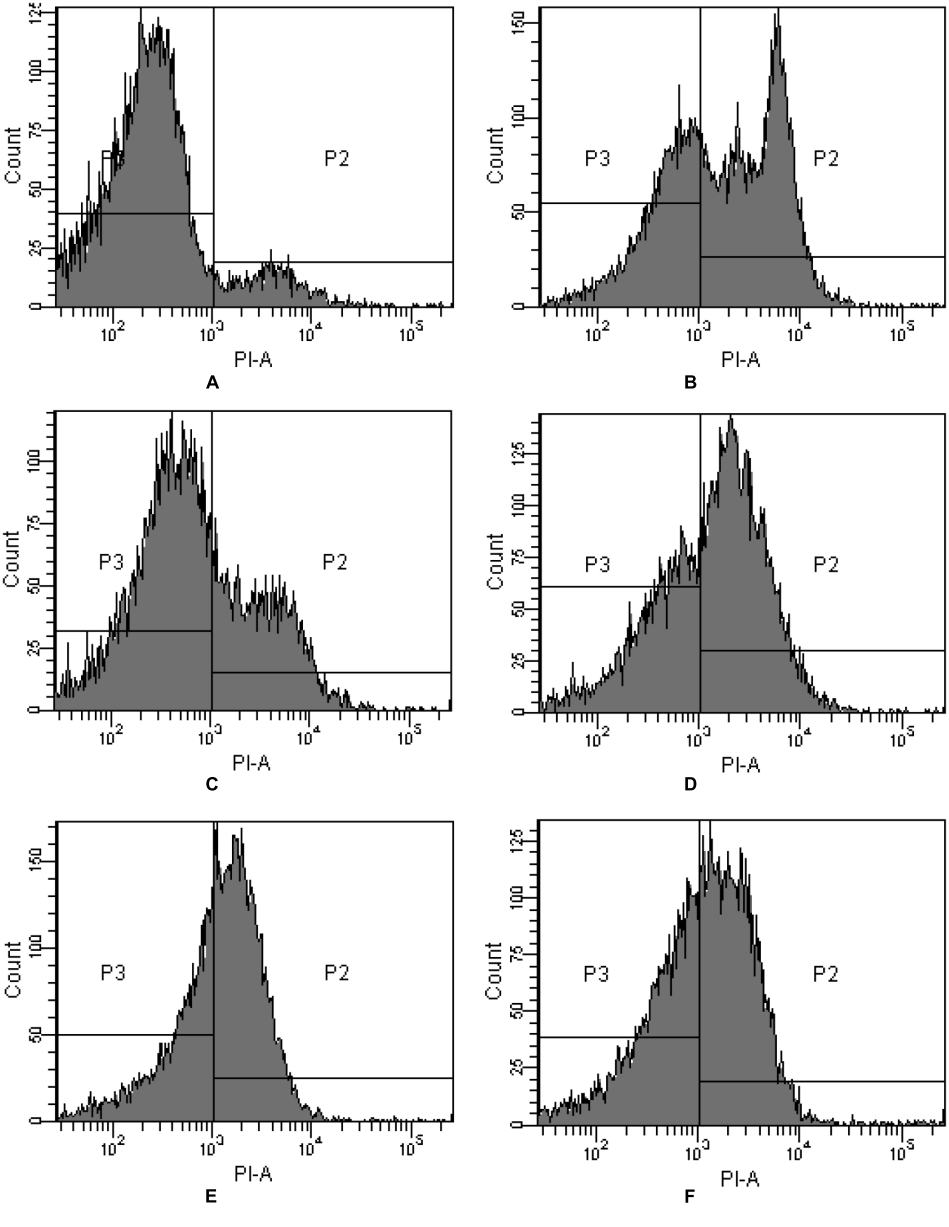
**Propidium iodide (PI) fluorescence density histogram of *P. globosa* in response to JS01 supernatant at different exposure time. (A)** Control, **(B)** 4 h, **(C)** 12 h, **(D)** 24 h, **(E)** 36 h, and **(F)** 48 h (quadrant P3: normal cells without PI fluorescence; quadrant P2: abnormal cells with PI fluorescence).

PI is commonly used for demonstrating membrane permeabilization. In fact it enters cells mainly via the damaged and permeabilized membranes, intercalating into DNA or RNA and producing a red fluorescence emission (Ritz et al., 2001). On comparing PI-stained algal cells with normal and membrane permeabilization cells, a large difference between the fluorescence was observed (**Figure 3**). The cytogram of control algal cells demonstrates that most of the algal cells were normal (**Figure 3A**), reaching a percentage of 91.4% (P3), while algal cells in P2 were permeabilized cells. Compared with the control, intracellular fluorescence increased significantly after 4 h exposure to JS01 supernatant and the percentage of permeabilized cells in P2 increased up to 59.3%, demonstrating significant damage to the algal cell membrane (**Figure 3B**). **Figures 3C–F** shows that PI fluorescence was significant higher in the permeabilized cells than in control cells. The percentages of permeabilized cells were 30.3, 59.2, 57.0, and 49.7% after 12, 24, 36, and 48 h treatment.

### Effect of ROS Levels, MDA Content and Antioxidative Enzyme Activity

**FIGURE 4:**
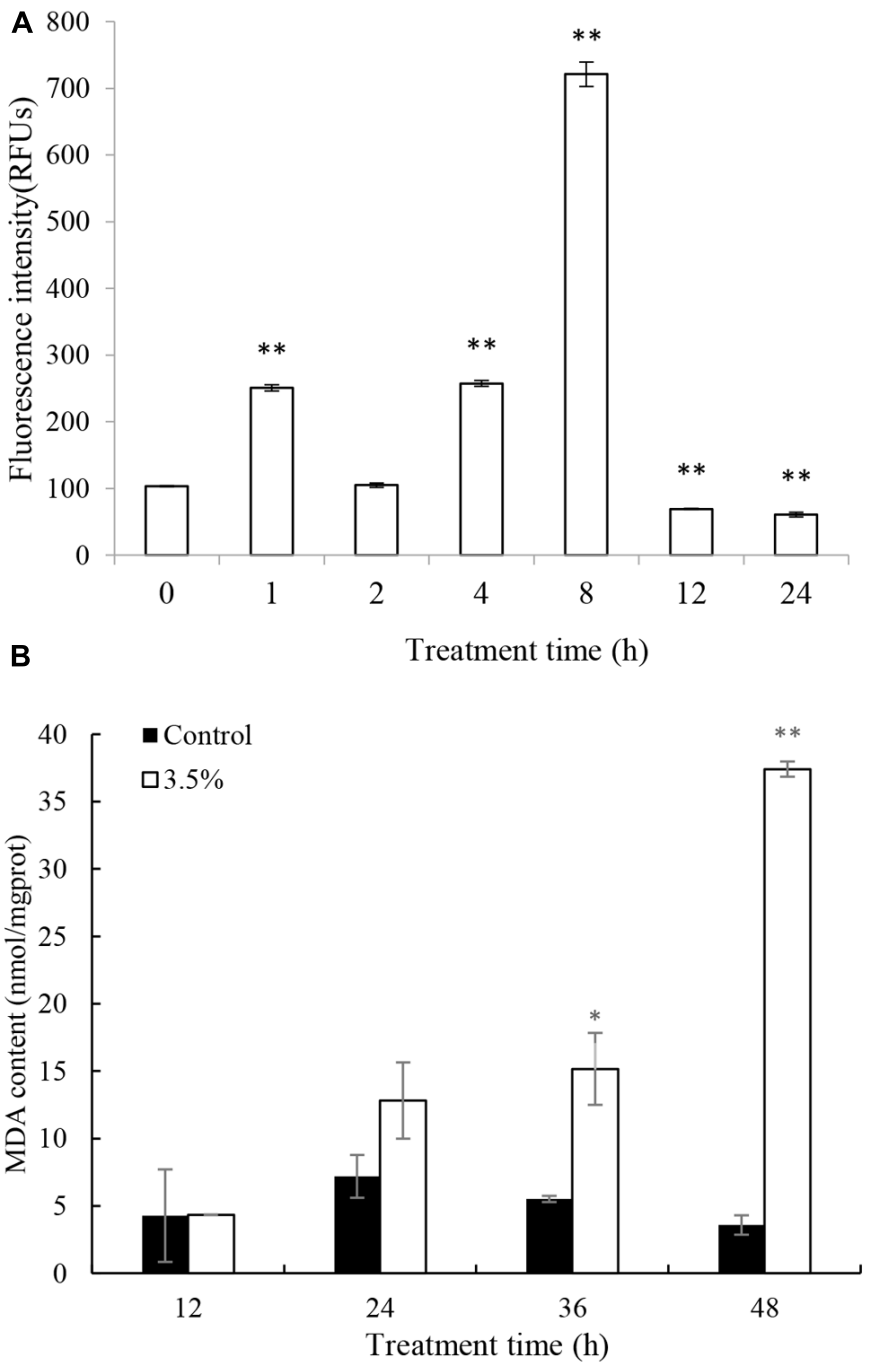
**Effects of JS01 supernatant on **(A)** ROS, **(B)** Malondialdehyde (MDA) contents of *P. globosa.*** All error bars indicate SE of the three replicates. ^∗^represents statistical significance at *p*< 0.05 and ^∗∗^ at *p*< 0.01.

Excessive ROS, generated during photosynthesis, is a strong oxidant that can potentially damage various molecules of biological importance. ROS production was assessed quantitatively by fluorescence intensity. The ROS production increased significantly (*p* < 0.05) after 1 h treatment and the level was 2.5 times that of 0 h (**Figure 4A**). After that, the ROS content decreased to a normal level by 2 h. However, the ROS content increased again after 4 h treatment and burst after 8 h treatment, accumulating to levels 2.5 and 7.2 times those of 0 h. The highest ROS level was at 8 h treatment and it decreased to a low level after 12 and 24 h treatment.

Malondialdehyde is a natural biomarker produced during membrane oxidative damage process(Qian et al., 2008). **Figure 4B** shows the content of MDA. Compared with the control, MDA contents in algal cells treated for 12 and 24 h increased slightly. As exposure time prolonged, MDA levels increased and the values were higher than the control group at the concentration of 3.5%. MDA levels were 2.75 (*p* < 0.05) and 10.44 (*p* < 0.01) times those of the control after treatment with JS01 supernatant for 36 and 48 h, respectively.

**FIGURE 5:**
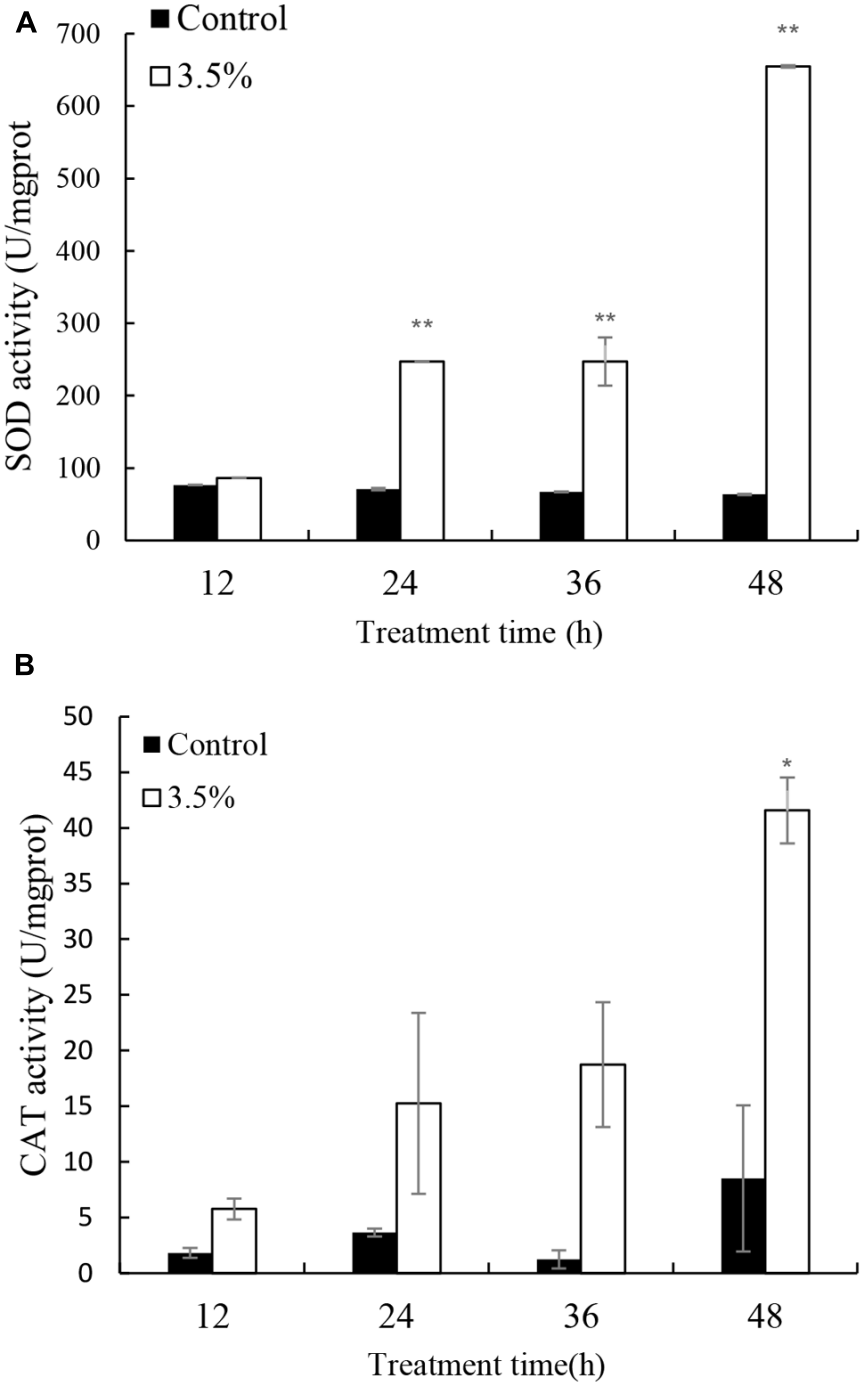
**Effects of JS01 supernatant on **(A)** SOD and **(B)** CAT contents of *P. globosa.*** All error bars indicate SE of the three replicates. ^∗^represents statistical significance at *p* < 0.05 and ^∗∗^ at *p* < 0.01.

Cellular enzyme activities including SOD and CAT were tested to investigate the response of antioxidant system induced by the JS01 supernatant (**Figure 5**). **Figure 5A** shows that the activities of SOD increased significantly compared with the control after algal cells were treated with JS01 supernatant. The activity values after 12, 24, 36, and 48 h were 1.13, 3.48 (*p* < 0.01), 3.69 (*p* < 0.01), and 10.31 times (*p* < 0.01) those of the control after exposure with 3.5% JS01 supernatant. The maximum SOD activity was at 48 h, showing that a longer exposure time could induce higher SOD activity in cells. CAT activity showed a similar trend with SOD activity, and the activity increased after 12 h treatment (**Figure 5B**). The maximum CAT activity was 4.88 times (*p* < 0.05) that of the control, which was observed at 48 h. The activity values after 12, 24, and 36 h treatment were 3.16, 4.67, and 14.87 times those of the control with 3.5% JS01 supernatant.

### Pigment Contents and Photosynthesis Efficiency Analysis

**FIGURE 6:**
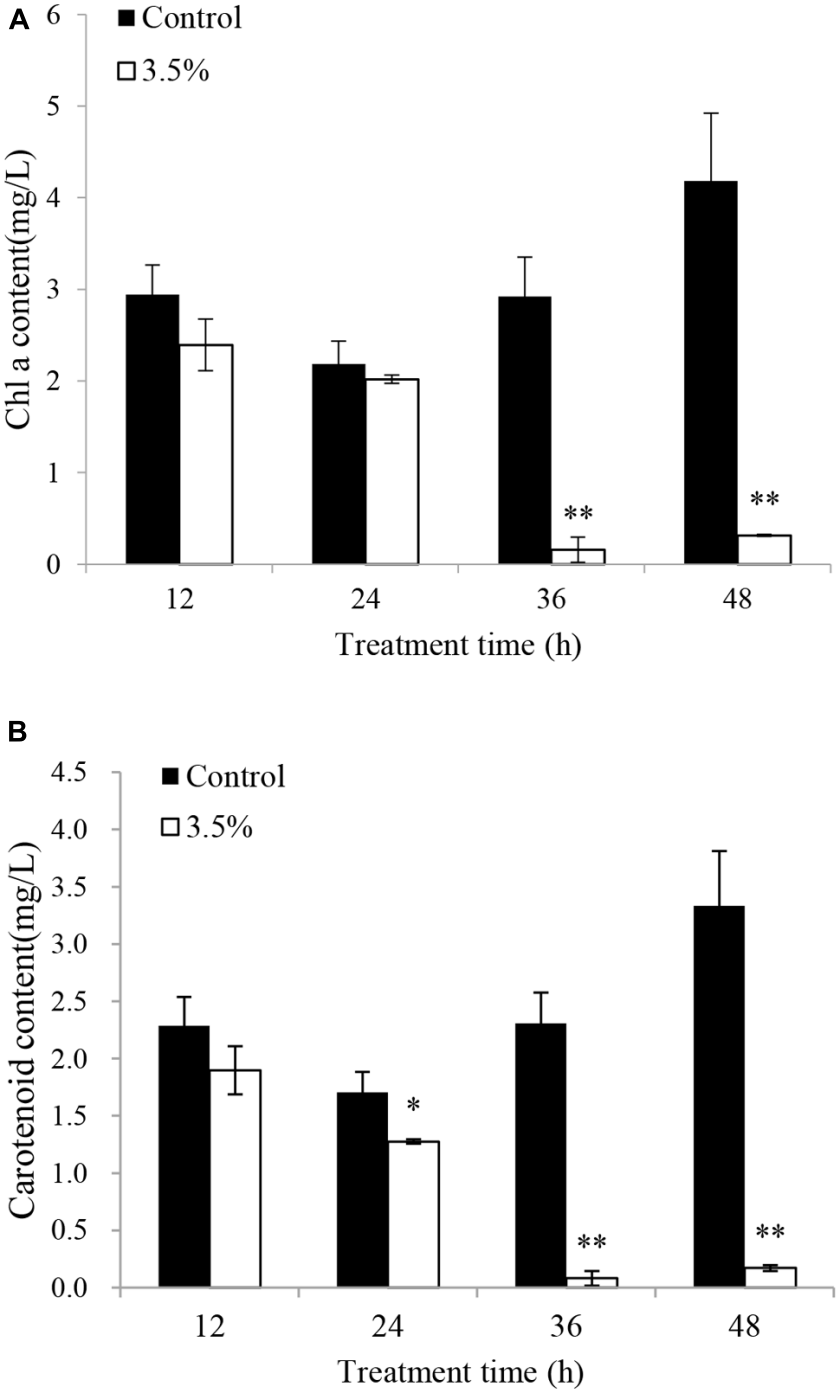
**Inhibitory effects of JS01 supernatant on **(A)** chlorophyll a and **(B)** carotenoid content in *P. globosa*.** All error bars indicate SE of the three replicates. ^∗^represents statistical significance at *p* < 0.05 and ^∗∗^ at *p* < 0.01.

The effects of JS01 supernatant on Chl *a* and carotenoid contents in *P. globosa* are shown in **Figure 6**. Compared with the control, Chl *a* contents were slightly lower than those of the control after 12 and 24 h exposure (**Figure 6A**). However, Chl *a* content decreased significantly (*p* < 0.01) when the treatment time was prolonged to 36 and 48 h, and the content of Chl *a* in the control cells was approximately 18.6 (*p* < 0.01) and 13.3 times (*p* < 0.01) those of treated cells after 36 and 48 h. **Figure 6B** indicates that the content of carotenoids in algal cells shared similar changes with the Chl *a* contents after treatment with 3.5% JS01 supernatant. After 24, 36, and 48 h, the contents of carotenoids in control cells were about 1.34 (*p* < 0.05), 28.66 (*p* < 0.01), and 19.49 times (*p* < 0.01) those of treatment cells.

**FIGURE 7:**
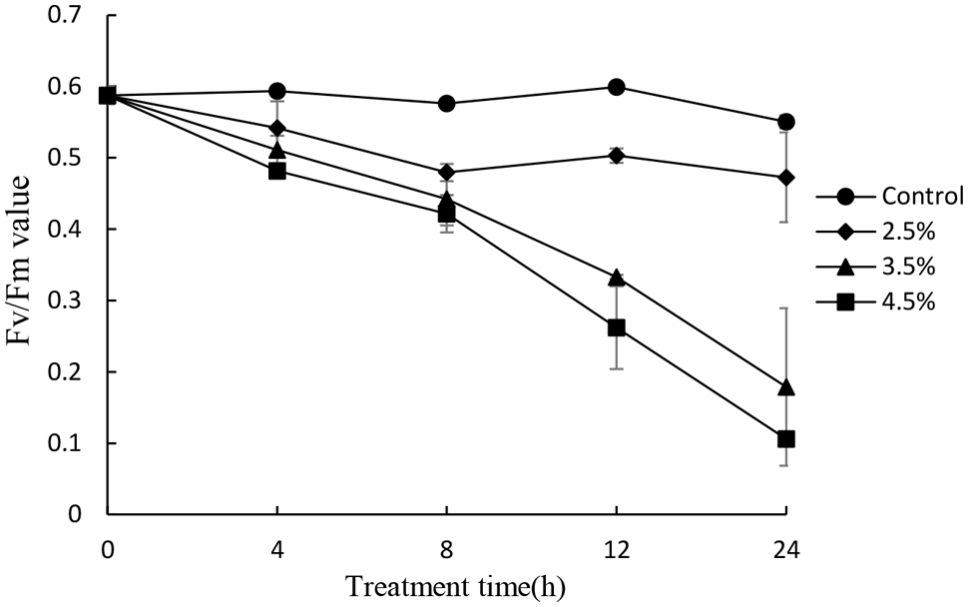
**Photosynthetic efficiency (Fv/Fm) of *P. globosa* cells treated with various concentrations of JS01 supernatant.** All error bars indicate SE of the three replicates.

To investigate the photosynthetic status of the cells under the stress of the JS01 supernatant, we studied the value of the maximum photochemical quantum yield (Fv/Fm) after treatment with the concentration of 2.5, 3.5, and 4.5% concentration of the JS01 supernatant. Within exposure in the concentration of 2.5% for 24h, the Fv/Fm ratio was slightly lower than those in control cells (**Figure 7**). We observed a lower Fv/Fm in the concentration of 3.5% treatment group. Moreover, the Fv/Fm values decreased significantly compared with the control in the 4.5% treatment group and this implied that the inhibition of the Fv/Fm in PS II was induced by JS01 supernatant.

### Involvement of the *psb*A and *rbc*S Genes in Response to JS01 Supernatant Stress

**FIGURE 8:**
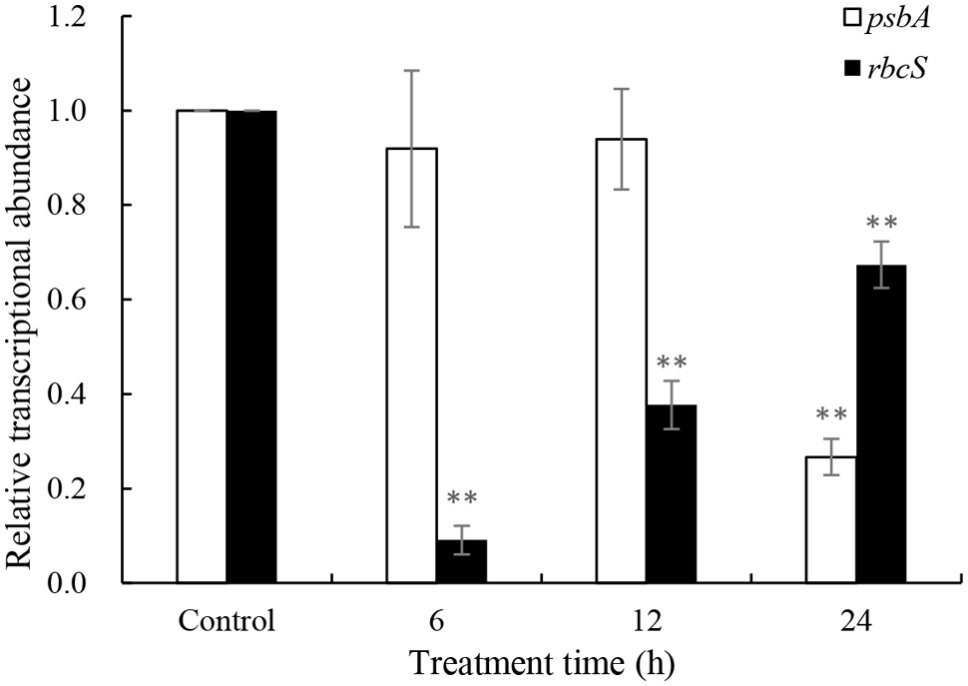
**Expression of *psbA* and *rbcS* genes in *P. globosa* exposed to JS01 supernatant.** Values were normalized to levels of 18S rRNA, a housekeeping gene, and represent the mean mRNA expression value ± SE (*n* = 3) relative to the control. ^∗∗^represents statistical significance at *p* < 0.01.

Repair of PS II from photodamage occurs by the *de novo* synthesis of reaction center D1 protein encoded by the *psbA* gene. To reveal whether the *psbA* respond to JS01 supernatant, expression of the *psbA* gene was analyzed using qRT-PCR in cells that were treated with 3.5% JS01 supernatant for 6, 12 and 24 h. As shown in **Figure 8**, the *psbA* gene was not so sensitive to JS01 supernatant in short time, but it decreased significantly (*p* < 0.01) when compared with the control after 24 h treatment, and the relative expression of *psbA* was 0.267 times that of the control. The *rbcS* gene is one of the best-known genes that encodes the small subunits of Rubisco which is an important enzyme for photosynthesis. However, the *rbcS* gene showed obviously different trends to the *psbA* gene (**Figure 8**). After 6 h exposure, it decreased significantly (*p* < 0.01) when compared with the control. Although the relative expressions of the *rbcS* gene increased as the exposure time was prolonged, it was also significantly (*p* < 0.01) lower than the control. Furthermore, the relative expression of *psbA* was 0.091, 0.377, and 0.674 times that of the control.

### Effect of JS01 Supernatant on the Subcellular Structure of *P. globosa*

**FIGURE 9:**
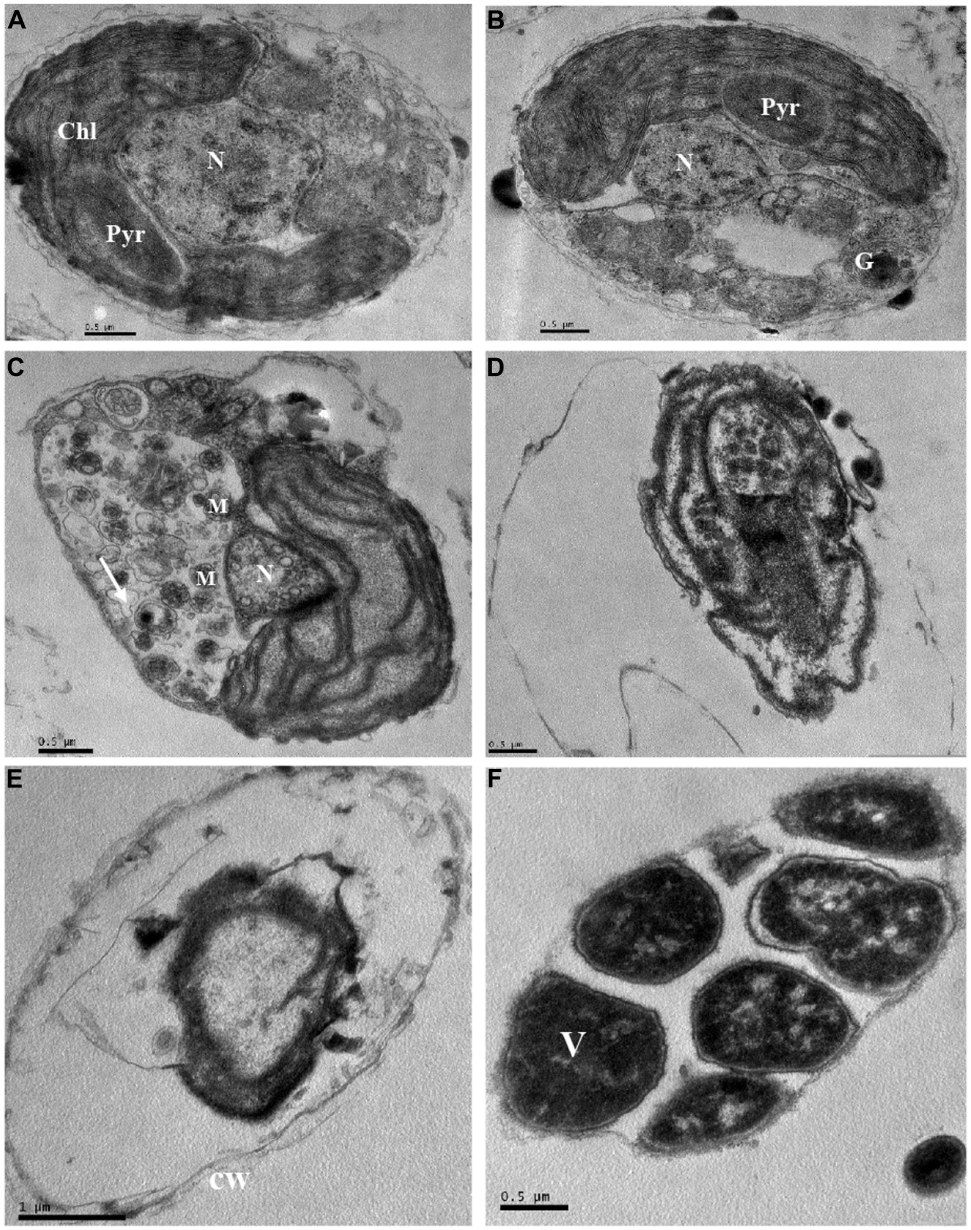
**Ultrastructure of *P. globosa* after exposure to JS01 supernatant with the concentration of 3.5% at different time. (A)** Control cell; **(B)** 12 h; **(C)** 24 h; **(D)** 36 h; **(E)** 48 h; **(F)** 72 h; N, nucleus; Chl, chloroplast; cw, cell wall; Pyr, pyrenoid; M, mitochondrion; G, Golgi body; V, vesicle; Bars **(A–F)** 0.5 μm; **(E)** 1 μm.

The ultrastructure of *P. globosa* was compared between control cells and those exposed to 3.5% JS01 supernatant for 12, 24, 36, 48, and 72 h (**Figure 9**). Compared with the control (**Figure 9A**), the JS01 supernatant treated cells showed many changes including morphological properties and some structural damage (**Figures 9B–F**). Algal cells treated for 12 and 24 h showed nucleus pyknosis and vacuolization. The number of multivesicular bodies was significantly increased and cell membrane was also destroyed (**Figure 9C**). The nucleus then became pyknotic, underwent karyorrhexis (fragmentation) and karyolysis (dissolution) after 36 h treatment (**Figures 9D–F**). Algal cells treated for 36 and 48 h showed extreme plasmolysis and vacuolization and many organelles such as the chloroplast, mitochondria and Golgi body were disorganized (**Figures 9D,E**). However, when algal cells were treated for 72 h, it was strange because many high density electric structures appeared in the cells and were arranged in a regular way.

## Discussion

The genus *Phaeocystis* is one of the most widespread marine phytoplankton taxa, and plays a significant role in global carbon and sulfur cycles, food web structure, and climate regulation, and it is also a harmful algal genus in coastal waters (Schoemann et al., 2005; Wang et al., 2010b). Bacteria and phytoplankton dynamics are thought to be closely linked in coastal marine environments, with correlations frequently observed between bacterial and phytoplankton biomass (Fuhrman et al., 1980). Several lines of evidence on HABs has revealed that marine bacteria are capable of promoting or inhibiting phytoplankton growth (Fukami et al., 1997; Iwata et al., 2004; Rooney-Varga et al., 2005). Cell lysis is an important loss factor in *P. globosa* blooms, as well as grazing (Sheik et al., 2013). However, it is indispensable to explore efficient materials to solve the blooms and study the mechanism of algal lysis process in the future.

*Phaeocystis globosa* could reach high biomass in nutritionally adequate waters after 18 days culture (**Figure 1**). It also indicated that it could grow rapidly in natural environment, especially in eutrophication marine waters. The testing of JS01 algicidal activity against *P. globosa* was performed using the Chl *a* removal rate. It seemed that the algicidal activity of JS01 was concentration dependent, since the supernatant concentration of the 1.5% group showed the lowest algicidal activity of the three groups (**Figure 2**). In addition, as the JS01 concentration increased, so the algicidal activity increased for a short time until 48 h because most of the algal cells were lysed at this time. Strain JS01 could produce red pigment when cultured in 2216E broth. A strain named MS-02-063, had been reported to produce a red pigment which belongs to prodigiosin members and showed potent algicidal activity against various red tide phytoplanktons (Nakashima et al., 2006). Moreover, we have sequenced the genome of strain JS01 and comparative genome analysis was also carried out now (Zhang et al., 2014). After that, we speculated that the algal effect of JS01 on *P. globose* may be correlates with one red pigment.

Flow cytometry detection PI uptake is a well-established and rapid method for monitoring cell death because PI could entry into cells when membrane permeability changed (Davey and Hexley, 2011). The FCM measurement of PI uptake (**Figure 3**) shows that when they were exposed to JS01 supernatant and stained with PI (**Figures 3B–F**), then a higher fraction of cells became PI-positive than did control cells. After 4 h exposure, 59.3% of the algal cells were PI-positive but the proportions of cells in each quadrant did not appear to have a defined trend as the treatment was prolonged. This indicated that the effect of JS01 on cells was not a physical disruption of cell structure to form debris, but a mechanism that causes the permeabilization of the cellular membrane, as demonstrated by the leakage of PI inside the permeabilized cells.

Environmental stresses can increase the generation of ROS, which can lead to severe cellular injury or death. Algal cell can produce high rates of ROS during electron flow process in chloroplast which can cause severely oxidative damage to algal cells (Perez-Perez et al., 2012). The indirect damage by ROS includes membrane oxidative, inhibition of photosynthesis, and oxidation of photosynthetic pigments such as chlorophylls and phycobilins (He and Häder, 2002). Our study demonstrated that JS01 supernatant induced a strong ROS formation in *P. globosa* (**Figure 4A**). It indicated that electron flow in chloroplast was severely inhibited, which induced a high production of ROS. Prolonged or acute stress under ROS may lead to irreversible damage to proteins, lipids, and nucleic acids (Oukarroum et al., 2012). MDA content can reflect cellular membrane oxidative damage status. As shown in **Figure 4B**, MDA contents increased after 24 h treatment and it increased significantly after 36 h (*p* < 0.05) and 48 h (*p* < 0.01) exposure. This test demonstrated that increasing ROS caused oxidative damage to the cellular membrane. Antioxidant enzymatic activities including SOD and CAT play important roles in scavenging ROS to protect algal cells. Our studies also proved that antioxidant enzymes in algal cells were triggered to eliminate excessive ROS when treated by JS01 supernatant (**Figure 5**). These results may indicate that ROS increased in algal cells under JS01 supernatant stress and antioxidant enzymes were activated to eliminate them.

Chl *a* is one of the primary light-harvesting pigment that play an important function in algal photosynthesis. In this study, a reduction in Chl *a* content was observed after exposure to JS01 supernatant from 12 to 48 h, and as time was prolonged the reduction was more obvious (**Figure 6A**). This indicated that the ability of cells to synthesize chlorophyll was decreased (Bornman and Vogelmann, 1991; Qian et al., 2009), and the lower chlorophyll content suggested a decrease in the antenna size of the photosynthetic reaction center complexes. Carotenoids are fundamental components of the photosynthetic tissues in plants, algae, and cyanobacteria, where they quench the excited triplet state of chlorophyll, preventing the formation of high levels of ROS to protect photosynthetic apparatus from photo-oxidative damage (Bartley and Scolnik, 1995; Vidhyavathi et al., 2008; Santabarbara et al., 2013). In our work, carotenoid content decreased as treatment time increased, especially after 36 and 48 h. This indicated that carotenoid synthesis was destroyed and the ability of algal cells to resist oxidative damage was also reduced. The PS II has been known to be very sensitive to changes in environment, and the extent of photoinactivation is a result of the balance between the photodamage to PS II and the repair of photodamaged PS II (Allakhverdiev, 2002; Takahashi and Badger, 2011). Photodamaged PS II is repaired by the replacement of damaged D1 protein by newly synthesized D1 (Murata et al., 2007), The decreased expression of *psb*A may destroy the balance between the damage to PS II and the repair of PS II and thus cause photosynthesis inhibition. RuBisCO is a key carbon fixation enzyme in plants and algae which is encoded by the *rbc*L and *rbc*S genes (Hwang and Tabita, 1991). Early observations suggest that suppression of the fixation of CO_2_ may enhance the extent of photoinhibition of PS II (Miller and Canvin, 1989; Murata et al., 2007) and expression of *rbc*L has a direct relationship with CO_2_ fixation. In our study, the *rbc*L gene decreased significantly (*p* < 0.01) during exposure time, especially after 6 h exposure. This implied that carbon fixation was also influenced and might induce damage to PS II. Fv/Fm is another indicator of the ability of photosynthesis process (**Figure 7**). Its value decreased as the concentration of the JS01 supernatant increased and with extension of the exposure time. These results indicated that PS II system did not have a normal photochemical reaction under external stress.

The intracellular ultrastructure of *P. globosa* has received little attention, and most studies are of colony morphology (Hamm, 2000; Peperzak et al., 2000; Peperzak and Gäbler-Schwarz, 2012). However, we observed obvious ultrastructure changes induced by the JS01 supernatant compared to normal cells (**Figure 9**). Algae cells experienced vacuolization under the stress of JS01 supernatant after 12 h exposure, and a pyrenoid also existed in chloroplast compared with control cell (**Figure 9A**). However, there were many multivesicular bodies in cells, and pyrenoids decomposed to provide more energy to resist external stress after 24 h exposure. The nucleus was even smaller than those in the control and 12 h treatment cells. Many organelles were disorganized and lost their function after 36 and 48 h exposure. Our interest was greatly promoted when algal cells were treated for 72 h because the electron dense deposits revealed in electron microscopy, at high magnification, were arranged regularly in an area. We supposed that it was a vesicle because *P. globosa* can form vesicles when it is under environmental stress and the outside wall is reinforced by algally secreted polysaccharide (van Rijssel et al., 1997; Schoemann et al., 2005).

## Conclusion

The results from our present study suggested that JS01 supernatant altered membrane permeability, enzymic antioxidant systems, pigment contents, and the photosynthesis process especially in terms of PS II and the subcellular structure in *P. globosa*. Our studies demonstrated that JS01 supernatant can change membrane permeability within a short time and then affect the photosynthesis process including gene expression, which might block the PS II electron transport chain and produce excessive ROS. The increased ROS can further change membrane permeability and destroy pigments, thus ultimately inducing algal cell death. And it also indicated that the algicidal actinomycete JS01 could function as an HAB controller material.

## Author Contributions

HZ contributes for conception and design, drafting of the article, technical and logistic support, analysis, and interpretation of the data. SZ and YP contribute for collection and assembly data and analysis the data. YL and ZC contribute for statistical expertise and collection and assembly data. HX contribute for critical revision of the article for important intellectual content. ZY and WZ contribute for obtaining of funding and provision of study materials. TZ contribute for obtaining of funding and final approval of the article. All authors had reviewed the manuscript.

## Conflict of Interest Statement

The authors declare that the research was conducted in the absence of any commercial or financial relationships that could be construed as a potential conflict of interest.

## Abbreviations

HABs: harmful algal blooms
ROS: reactive oxygen species
PS II: photosystem II
SOD: superoxide dismutase
CAT: catalase
FCM: flow cytometry

## References

[B1] Allakhverdiev S. I. (2002). Salt stress inhibits the repair of photodamaged photosystem ii by suppressing the transcription and translation of psbA genes in synechocystis. *Plant Physiol.* 130 1443–1453. 10.1104/pp.011114 12428009 PMC166663

[B2] Anderson D. M. (1997). Turning back the harmful red tide. *Nature* 388 513–514. 10.1038/41415

[B3] Anderson D. M. Cembella A. D. Hallegraeff G. M. (2012). Progress in understanding harmful algal blooms: paradigm shifts and new technologies for research, monitoring, and management. *Annu. Rev. Mar. Sci.* 4 143–176. 10.1146/annurev-marine-120308-081121 PMC5373096 22457972

[B4] Bartley G. E. Scolnik P. A. (1995). Plant carotenoids: pigments for photoprotection, visual attraction, and human health. *Plant Cell* 7 1027–1038. 10.1105/tpc.7.7.1027 7640523 PMC160905

[B5] Baudoux A. C. Brussaard C. P. (2005). Characterization of different viruses infecting the marine harmful algal bloom species *Phaeocystis globosa*. *Virology* 341 80–90. 10.1016/j.virol.2005.07.002 16081120

[B6] Bornman J. F. Vogelmann T. C. (1991). Effect of UV-B radiation on leaf optical properties measured with fibre optics. *J. Exp. Bot.* 42 547–554. 10.1093/jxb/42.4.547

[B7] Brussaard C. P. D. Mari X. Bleijswijk J. D. L. V. Veldhuis M. J. W. (2005). A mesocosm study of *Phaeocystis globosa* (Prymnesiophyceae) population dynamics. *Harmful Algae* 4 875–893. 10.1016/j.hal.2004.12.012

[B8] Chen Z. Lei X. Zhang B. Yang L. Zhang H. Zhang J. (2014). First report of *Pseudobodo* sp, a new pathogen for a potential energy-producing algae: *Chlorella vulgaris* cultures. *PLoS ONE* 9:e89571 10.1371/journal.pone.0089571 PMC3943784 24599263

[B9] Choi H. Kim B. Kim J. Han M. (2005). Streptomyces neyagawaensis as a control for the hazardous biomass of *Microcystis aeruginosa* (Cyanobacteria) in eutrophic freshwaters. *Biol. Control* 33 335–343. 10.1016/j.biocontrol.2005.03.007

[B10] Davey H. M. Hexley P. (2011). Red but not dead? Membranes of stressed *Saccharomyces cerevisiae* are permeable to propidium iodide. *Environ. Microbiol.* 13 163–171. 10.1111/j.1462-2920.2010.02317.x 21199254

[B11] DiTullio G. Grebmeier J. Arrigo K. Lizotte M. Robinson D. Leventer A. (2000). Rapid and early export of *Phaeocystis* antarctica blooms in the Ross Sea, Antarctica. *Nature* 404 595–598. 10.1038/35007061 10766240

[B12] Drábková M. Admiraal W. Maršálek B. (2007). Combined exposure to hydrogen peroxide and light selective effects on cyanobacteria, green algae, and diatoms. *Environ. Sci. Technol.* 41 309–314. 10.1021/es060746i 17265964

[B13] Faber M. J. Smith L. M. Boermans H. J. Stephenson G. R. Thompson D. G. Solomon K. R. (1997). Cryopreservation of fluorescent marker-labeled algae (*Selenastrum capricornutum*) for toxicity testing using flow cytometry. *Environ. Toxicol. Chem.* 16 1059–1067. 10.1002/etc.5620160528

[B14] Fuhrman J. A. Ammerman J. W. Azam F. (1980). Bacterioplankton in the coastal euphotic zone: distribution, activity and possible relationships with phytoplankton. *Mar. Biol.* 60 201–207. 10.1007/BF00389163

[B15] Fukami K. Nishijima T. Ishida Y. (1997). “Stimulative and inhibitory effects of bacteria on the growth of microalgae,” in Live Food in Aquaculture. *Springer* 124 185–191.

[B16] Hamm C. E. (2000). Architecture, ecology and biogeochemistry of *Phaeocystis* colonies. *J. Sea Res.* 43 307–315. 10.1016/S1385-1101(00)00014-9

[B17] He Y. Y. Häder D. P. (2002). Involvement of reactive oxygen species in the UV-B damage to the cyanobacterium *Anabaena* sp. *J. Photochem. Photobiol. B Biol.* 66 73–80. 10.1016/S1011-1344(01)00278-0 11849986

[B18] Hoogstraten A. Peters M. Timmermans K. R. De Baar H. J. W. (2012). Combined effects of inorganic carbon and light on *Phaeocystis globosa* Scherffel (Prymnesiophyceae). *Biogeosciences* 9 1885–1896. 10.5194/bg-9-1885-2012

[B19] Hwang S.-R. Tabita F. (1991). Cotranscription, deduced primary structure, and expression of the chloroplast-encoded rbcL and rbcS genes of the marine diatom *Cylindrotheca* sp. strain N1. *J. Biol. Chem.* 266 6271–6279. 1706714

[B20] Inskeep W. P. Bloom P. R. (1985). Extinction coefficients of chlorophyll a and b in N, N-dimethylformamide and 80% acetone. *Plant Physiol.* 77 483–485. 10.1104/pp.77.2.483 16664080 PMC1064541

[B21] Iwata Y. Sugahara I. Kimura T. Kowa H. Matsumoto A. Noritake K. (2004). Properties of an algicidal bacterium (*Flavobacterium* sp.) against Karenia mikimotoi isolated from Ise Bay, Japan. *Nippon Suisan Gakkaishi* 70 537–541. 10.2331/suisan.70.537

[B22] Lamy D. Obernosterer I. Laghdass M. Artigas F. Breton E. Grattepanche J. D. (2009). Temporal changes of major bacterial groups and bacterial heterotrophic activity during a *Phaeocystis globosa* bloom in the eastern English Channel. *Aquat. Microbial. Ecol.* 58 95–107. 10.3354/ame01359

[B23] Leitão M. A. S. Cardozo K. Pinto E. Colepicolo P. (2003). PCB-induced oxidative stress in the unicellular marine dinoflagellate Lingulodinium polyedrum. *Arch. Environ. Contam. Toxicol.* 45 59–65. 10.1007/s00244-002-0208-5 12948173

[B24] Li D. Zhang H. Fu L. An X. Zhang B. Li Y. (2014a). A novel algicide: evidence of the effect of a fatty acid compound from the marine bacterium, *Vibrio* sp. BS02 on the harmful dinoflagellate, *Alexandrium tamarense*. *PLoS ONE* 9:e91201 10.1371/journal.pone.0091201 PMC3953379 24626054

[B25] Li Y. Zhu H. Guan C. Zhang H. Guo J. Chen Z. (2014b). Towards molecular, physiological, and biochemical understanding of photosynthetic inhibition and oxidative stress in the toxic *Alexandrium tamarense* induced by a marine bacterium. *Appl. Microbiol. Biotechnol.* 98 4637–4652. 10.1007/s00253-014-5578-x 24682476

[B26] Liu W. Chen S. Quan X. Jin Y. H. (2008). Toxic effect of serial perfluorosulfonic and perfluorocarboxylic acids on the membrane system of a freshwater alga measured by flow cytometry. *Environ. Toxicol. Chem.* 27 1597–1604. 10.1897/07-459 18269298

[B27] Livak K. J. Schmittgen T. D. (2001). Analysis of relative gene expression data using real-time quantitative PCR and the 2-ΔΔct method. *Methods* 25 402–408. 10.1006/meth.2001.1262 11846609

[B28] Miller A. G. Canvin D. T. (1989). Glycolaldehyde inhibits CO2 fixation in the cyanobacterium *Synechococcus* UTEX 625 without inhibiting the accumulation of inorganic carbon or the associated quenching of chlorophyll a fluorescence. *Plant Physiol.* 91 1044–1049. 10.1104/pp.91.3.1044 16667109 PMC1062116

[B29] Murata N. Takahashi S. Nishiyama Y. Allakhverdiev S. I. (2007). Photoinhibition of photosystem II under environmental stress. *Biochim. Biophys. Acta* 1767 414–421. 10.1016/j.bbabio.2006.11.019 17207454

[B30] Nakashima T. Miyazaki Y. Matsuyama Y. Muraoka W. Yamaguchi K. Oda T. (2006). Producing mechanism of an algicidal compound against red tide phytoplankton in a marine bacterium γ-proteobacterium. *Appl. Microbiol. Biotechnol.* 73 684–690. 10.1007/s00253-006-0507-2 16850298

[B31] Oukarroum A. Bras S. Perreault F. Popovic R. (2012). Inhibitory effects of silver nanoparticles in two green algae, *Chlorella vulgaris* and *Dunaliella tertiolecta*. *Ecotoxicol. Environ. Saf.* 78 80–85. 10.1016/j.ecoenv.2011.11.012 22138148

[B32] Peperzak L. Colijn F. Vrieling E. Gieskes W. Peeters J. (2000). Observations of flagellates in colonies of *Phaeocystis globosa* (Prymnesiophyceae); a hypothesis for their position in the life cycle. *J. Plankton Res.* 22 2181–2203. 10.1093/plankt/22.12.2181

[B33] Peperzak L. Gäbler-Schwarz S. (2012). Current knowledge of the life cycles of *Phaeocystis globosa* and *Phaeocystis* antarctica (prymnesiophyceae). *J. Phycol.* 48 514–517. 10.1111/j.1529-8817.2012.01136.x 27011066

[B34] Perez-Perez M. E. Lemaire S. D. Crespo J. L. (2012). Reactive oxygen species and autophagy in plants and algae. *Plant Physiol.* 160 156–164. 10.1104/pp.112.199992 22744983 PMC3440194

[B35] Qian H. Chen W. Sheng G. D. Xu X. Liu W. Fu Z. (2008). Effects of glufosinate on antioxidant enzymes, subcellular structure, and gene expression in the unicellular green alga *Chlorella vulgaris*. *Aquat. Toxicol.* 88 301–307. 10.1016/j.aquatox.2008.05.009 18584892

[B36] Qian H. Xu X. Chen W. Jiang H. Jin Y. Liu W. (2009). Allelochemical stress causes oxidative damage and inhibition of photosynthesis in *Chlorella vulgaris*. *Chemosphere* 75 368–375. 10.1016/j.chemosphere.2008.12.040 19171365

[B37] Ritz M. Tholozan J. Federighi M. Pilet M. (2001). Morphological and physiological characterization of *Listeria monocytogenes* subjected to high hydrostatic pressure. *Appl. Environ. Microbiol.* 67 2240–2247. 10.1128/AEM.67.5.2240-2247.2001 11319107 PMC92862

[B38] Rooney-Varga J. N. Giewat M. W. Savin M. C. Sood S. Legresley M. Martin J. (2005). Links between phytoplankton and bacterial community dynamics in a coastal marine environment. *Microb. Ecol.* 49 163–175. 10.1007/s00248-003-1057-0 15688258

[B39] Santabarbara S. Casazza A. P. Ali K. Economou C. K. Wannathong T. Zito F. (2013). The requirement for carotenoids in the assembly and function of the photosynthetic complexes in *Chlamydomonas reinhardtii*. *Plant Physiol.* 161 535–546. 10.1104/pp.112.205260 23161889 PMC3532283

[B40] Santini S. Jeudy S. Bartoli J. Poirot O. Lescot M. Abergel C. (2013). Genome of *Phaeocystis globosa* virus PgV-16T highlights the common ancestry of the largest known DNA viruses infecting eukaryotes. *Proc. Natl. Acad. Sci. U.S.A.* 110 10800–10805. 10.1073/pnas.1303251110 23754393 PMC3696832

[B41] Schoemann V. Becquevort S. Stefels J. Rousseau V. Lancelot C. (2005). Phaeocystis blooms in the global ocean and their controlling mechanisms: a review. *J. Sea Res.* 53 43–66. 10.1016/j.seares.2004.01.008

[B42] Sheik A. R. Brussaard C. P. Lavik G. Foster R. A. Musat N. Adam B. (2013). Viral infection of *Phaeocystis globosa* impedes release of chitinous star-like structures: quantification using single cell approaches. *Environ. Microbiol.* 15 1441–1451. 10.1111/j.1462-2920.2012.02838.x 22857133

[B43] Spilimbergo S. Foladori P. Mantoan D. Ziglio G. Della Mea G. (2010). High-pressure CO2 inactivation and induced damage on *Saccharomyces cerevisiae* evaluated by flow cytometry. *Process Biochem.* 45 647–654. 10.1016/j.procbio.2009.12.013

[B44] Su J. Yang X. Zheng T. Hong H. (2007). An efficient method to obtain axenic cultures of *Alexandrium tamarense*—a PSP-producing dinoflagellate. *J. Microbiol. Methods* 69 425–430. 10.1016/j.mimet.2006.07.005 17307263

[B45] Takahashi S. Badger M. R. (2011). Photoprotection in plants: a new light on photosystem II damage. *Trends Plant Sci.* 16 53–60. 10.1016/j.tplants.2010.10.001 21050798

[B46] van Rijssel M. Hamm C. Gieskes W. (1997). *Phaeocystis globosa* (Prymnesiophyceae) colonies: hollow structures built with small amounts of polysaccharides. *Eur. J. Phycol.* 32 185–192. 10.1017/S0967026297001108

[B47] Vidhyavathi R. Venkatachalam L. Sarada R. Ravishankar G. A. (2008). Regulation of carotenoid biosynthetic genes expression and carotenoid accumulation in the green alga *Haematococcus pluvialis* under nutrient stress conditions. *J. Exp. Bot.* 59 1409–1418. 10.1093/jxb/ern048 18343887

[B48] Wang B. X. Zhou Y. Y. Bai S. J. Su J. Q. Tian Y. Zheng T. L. (2010a). A novel marine bacterium algicidal to the toxic dinoflagellate *Alexandrium tamarense*. *Lett. Appl. Microbiol.* 51 552–557. 10.1111/j.1472-765X.2010.02936.x 20880149

[B49] Wang X. Tang K. W. Wang Y. Smith W. O. (2010b). Temperature effects on growth, colony development and carbon partitioning in three *Phaeocystis* species. *Aquat. Biol.* 9 239–249. 10.3354/ab00256

[B50] Yang C.-Y. Liu S. J. Zhou S. W. Wu H. F. Yu J. B. Xia C. H. (2011). Allelochemical ethyl 2-methyl acetoacetate (EMA) induces oxidative damage and antioxidant responses in *Phaeodactylum tricornutum*. *Pestic. Biochem. Physiol.* 100 93–103. 10.1016/j.pestbp.2011.02.014

[B51] Yin L. Huang J. Huang W. Li D. Wang G. Liu Y. (2005). Microcystin-RR-induced accumulation of reactive oxygen species and alteration of antioxidant systems in tobacco BY-2 cells. *Toxicon* 46 507–512. 10.1016/j.toxicon.2005.06.015 16084553

[B52] Zhang H. An X. Zhou Y. Zhang B. Zhang S. Li D. (2013a). Effect of oxidative stress induced by *Brevibacterium* sp. BS01 on a HAB causing species-*Alexandrium tamarense*. *PLoS ONE* 8:e63018 10.1371/journal.pone.0063018 PMC3648478 23667564

[B53] Zhang Y. Chen H. He C. Wang Q. (2013b). Nitrogen starvation induced oxidative stress in an oil-producing green alga *Chlorella sorokiniana* C3. *PLoS ONE* 8:e69225 10.1371/journal.pone.0069225 PMC3712941 23874918

[B54] Zhang H. Zhang S. Peng Y. Li Y. Chen Z. Zheng W. (2014). Draft genome sequence of the anti-algal marine actinomycete *Streptomyces* sp. JS01. *Genome Announc.* 2 e01261–e01214 10.1128/genomeA.01261-14 PMC4256195 25477414

